# Porous sulfur polymers for effective aqueous-phase organic contaminant removal

**DOI:** 10.1038/s41598-024-57856-8

**Published:** 2024-04-07

**Authors:** Vinicius Diniz, Joseph C. Bear, Susanne Rath, Colin R. Crick

**Affiliations:** 1 School of Engineering and Materials Sciences, Queen Mary University of London, London, E1 4NS UK; 2 Institute of Chemistry, University of Campinas, Campinas, 13083-970 Brazil; 3 School of Life Sciences, Pharmacy and Chemistry, Kingston University, Penrhyn Road, Kingston-Upon-Thames, KT1 2EE UK

**Keywords:** Adsorption, Caffeine, Inverse vulcanization, Photocatalysis, Porous materials, Nanocomposites, Polymer characterization, Polymer synthesis, Pollution remediation, Environmental chemistry

## Abstract

Sulfur polymers produced through 'inverse vulcanization' exhibit various attributes, such as photocatalytic activity and a high capacity to adsorb heavy metals. Nevertheless, there is a lack of research investigating the use of sulfur polymers as materials for the removal of organic contaminants. In this work, porous sulfur polymers (PSPs) were synthesized from elemental sulfur and 1,3-diisopropenylbenzene, with porosity introduced via salt templating. The result is a material that can strongly adsorb and chemically neutralize a model organic contaminant (caffeine). PSPs show adsorption up to 5 times higher than a leading adsorption material (activated carbon). Furthermore, either the adsorption or degradation processes can govern the removal efficiency depending on the synthesis parameters of PSPs. This is the first-ever report demonstrating sulfur polymers as effective materials for removing emerging contaminants from water. The versatile synthesis of sulfur polymers offers variation, which means that there is much more to explore in this exciting research area.

## Introduction

Over the past 50 years, global freshwater use surged by 121%, while resources dropped by 52.1% (Figure S1)^1^. The United Nations 2030 Agenda outlines seventeen goals, including making cities resilient, ensuring water access, and fostering sustainable industries. Direct potable reuse (DPR) and, in coastal areas, seawater desalination emerging as promising alternatives to deal with water scarcity due to their capability of reducing vulnerability by increasing resilience, diversity, adaptability, and sustainability of drinking-water supplies, developing new and preferably more climate independent water resources in close proximity to major population centres^2^. However, seawater desalination is generally restricted to coastal areas^2^, which means that DPR is an interesting alternative to produce large volumes of drinking-water from wastewater in both coastal and inland locations^3^. According to the World Health Organization, a DRP scheme requires performance with four Rs (reliability, redundancy, robustness, and resilience)^2^, which means the implementation of multi-barrier (membrane filtration, advanced oxidation, and media filtration units) water treatment processes, which can be costly compared to conventional treatment processes, such activated sludge or coagulation and flocculation over the years, as can be seen in Figure S2^4^.

Elemental sulfur (S_8_) is a by-product of the hydrodesulfurization process used to remove sulfur-containing compounds from petroleum during refinement, and is estimated to be produced at more than 60 million tons each year^5^. Given the relative abundance and affordability of sulfur, it provides a highly promising area to explore new approaches and techniques for creating innovative functional materials^6^. One such technique is “inverse vulcanization”, which is a solvent-free process introduced by Chung et al*.* in 2013^5^. By incorporating unsaturated organic monomers with elemental sulfur, this process enables the production of polymers with high sulfur content, usually at elevated temperatures^7^. The added monomer stabilizes sulfur chains using crosslinking, forming a hyperbranched network, which imparts stability against depolymerization and results in the creation of durable and functional materials^6,8^.

“Inverse vulcanization” sulfur polymers have a wide range of applications in different fields such as optical, optoelectronic, superhydrophobic materials, and photochemical materials, along with pharmaceutical preparations based on biopolymers, proton-conducting electrolytes, cathodes in lithium-sulfur batteries, and electromembrane processes^8–11^. Amongst these applications, the use of porous sulfur-containing polymers (PSPs) for the gas phase adsorption of heavy metals, such as mercury, has already been proposed^6,12,13^. Additionally, Upton et al*.*^8^ reported photoactive properties of sulfur-containing polymers when irradiated by UV-C lamps (254 nm), as well as antibacterial properties. Although these materials have been used in a range of fields, (*e.g.* antifouling, or heavy metal removal) to the best of our knowledge, no studies have been performed to evaluate the potential of these polymers to be used for removing organic contaminants from aqueous matrices, such as emerging contaminants (pharmaceuticals, personal care products, and endocrine disruptors). Caffeine was chosen as the representative compound for our removal studies. This selection was based on the reported presence of caffeine in water bodies, with concentrations ranging from ng L^−1^ to µg L^−1^, as documented in previous studies^14–16^ and its use as tracer for anthropogenic contamination^17^. Furthermore, the effectiveness of caffeine removal by wastewater treatment plants and drinking water treatment plants can differ significantly based on the technologies employed, leading to persistently high concentrations in the effluent, as noted in previous research^14,18^.

Although the potable water generation process is established^19^, challenges persist in enhancing efficiency, cost-effectiveness, and sustainability, speeding up future innovations^20^. Among the different materials that have been proposed in the literature, “inverse vulcanisation” sulfur polymers are inexpensive and relatively easy to synthesize^13,21^, and with the use of table salt as a template to create pores have been used for the adsorption of mercury and other heavy metals^6,12,22^ from both air and water phase and gas selectivity^13^. In this present study, we demonstrate that PSPs (here, synthesized via “inverse vulcanization”, using 1,3-diisopropenylbenzene (DIB)) can also be used for aqueous phase-adsorption and degradation of organic contaminants, adding a new potential application of these materials. Further, the properties of the PSPs produced simply by the removal of the template, such as effective adsorption and photocatalysis, are dependent on the ratio of sulfur and DIB from which they are formed.

## Materials and methods

### Chemicals and reagents

Elemental sulfur (S_8_, sublimed powder, reagent grade, ≥ 99.5%) was purchased from Honeywell Lab (UK). 1,3-diisopropenylbenzene (stabilized with TBC, > 97%) was purchased from TCI Limited. Caffeine (99.0%) was purchased from Sigma-Aldrich (UK). Deuterated chloroform (D, 99.8%) was purchased from Cambridge Isotopes Laboratories (UK). “A pinch of table salt” [Brand Name] as the source of NaCl was obtained from a Co-op (London, UK). Ethanol (p.a), hydrochloric acid (35%) and sodium hydroxide (p.a.) were purchased from Fischer Chemical (UK).

### Synthesis of porous sulfur polymers

**Figure 1:**
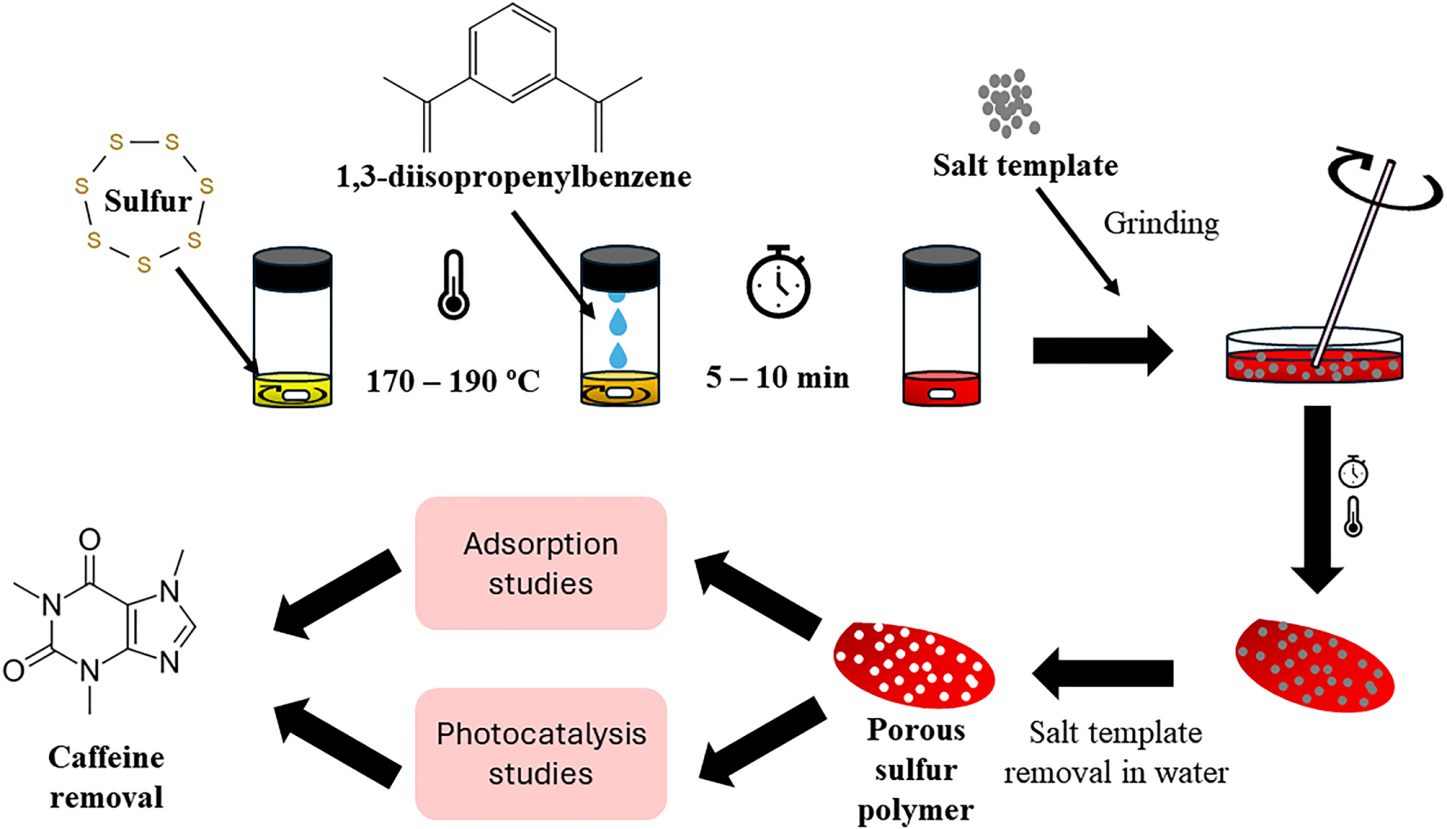
General schematic diagram for the preparation and application of the porous sulfur polymers.

A general schematic diagram for the preparation of PSPs is shown in Fig. 1. Briefly, elemental sulfur was heated to ca. 170–190 °C under continuous magnetic stirring. The chosen temperature range has been carefully determined to facilitate the opening of elemental sulfur rings^8^, while simultaneously preventing the degradation or breakdown of the elemental sulfur molecules. Once completely molten (indicated by a change in appearance from a pale-yellow powder to a yellow/orange liquid), DIB was gradually added dropwise. In the experiments, the elemental sulfur:DIB monomer mass ratio was varied from 40:60 to 90:10. DIB content higher than 40% led to non-rigid, sticky PSPs. Conversely, 100% sulfur PSPs are brittle^6^. The mixtures were stirred at 1500 rpm for 5–10 min until the reaction was nearly complete. Subsequently, the still-liquid pre-polymer was transferred into a silicone mould containing different amounts of table salt (ground using a pestle and mortar, with ratios of 1:4 to 3:2 (mass of table salt/mass of sulfur)). The mixture was thoroughly blended with a glass rod and left to cure at 140 °C for 4 h. For the template removal, the PSPs were stirred with ultrapure water overnight, filtered and then dried. The PSPs were named according to the amount of DIB, sulfur and table salt used. For example, the PSP synthesized with 40%[DIB]:60%[S_8_]:1.000 g[NaCl] is named PSP_40:60:1.000._

### Characterization of porous sulfur polymers

The characterization of PSPs encompassed a series of analytical techniques. Nitrogen adsorption/desorption isotherms were acquired at 77 K using a surface area analyzer (NOVA 4200e, Quantachrome). Prior to analysis, the materials underwent a 24-h degassing process under vacuum at 35 °C. Scanning Electron Microscopy (SEM) imaging and Energy-Dispersive X-ray Spectroscopy (EDS) were performed using an FEI Inspect F system with an operational acceleration voltage of 10–20 kV. To enhance electrical conductivity within the SEM, samples were sputter-coated with a thin layer of gold using an Automatic Sputter Coater. Fourier transformed infrared (FTIR) spectra were recorded using a Bruker Tensor 27 instrument over the wavenumber range of 500 to 4000 cm^−1^. Nuclear magnetic resonance (NMR) analysis utilized a Bruker Advance DRX (400 MHz) spectrometer, with deuterated chloroform as the solvent and tetramethylsilane as the internal standard. All NMR spectra were obtained at room temperature. Differential scanning calorimetry (DSC) measurements were carried out using a TA Instruments Discovery Series DSC 25. A heat-cool-heat method was employed, with heating and cooling rates set at 10 °C min^−1^, spanning from − 20 to 150 °C. Powder X-ray diffraction (patterns were collected in reflection mode using a Panalytical X’Pert PRO MPD equipped with a high throughput screening XYZ stage, X-ray focusing mirror, and PIXcel detector. Cu Kα radiation was utilized, and data were collected over a range of 5–70° using loose powder samples on thin Mylar film within aluminium well plates. Thermogravimetric analysis (TGA) (was conducted under an inert atmosphere on a TA Instruments TGA 5500. Heating was carried out at a heating rate of 10 ºC min^−1^, from room temperature to 600 °C. The investigation of the point of zero charge (PZC) of the PSPs was determined according to Diniz et al*.*^14^. Briefly, a 300 mL solution containing 0.01 mol L^−1^ NaCl was evenly distributed into 10 mL glass vials (10 mL per vial). The pH of each vial was carefully adjusted with HCl or NaOH within the range of 0.5 to 11.5. Subsequently, 50 mg of PSPs was introduced into each vial. These mixtures were then maintained at a temperature of 25 °C for 48 h to assess the resultant pH values.

### Removal experiments

#### Adsorption studies

The batch adsorption experiments were conducted following the guidelines of the American Society for Testing and Materials (ASTM) protocol D3860/2020. A total of 400 mL of water, containing 7.5 mg L^−1^ of caffeine, was used in a 500 mL Beaker. To prepare the standards, low concentrations of methanol (< 0.5% v/v) were employed due to the low solubility of caffeine^14^. The studies were performed at the mg L^−1^ concentration range to facilitate monitoring and reduce any uncertainty in the results obtained^18,23–26^.

For the adsorption studies, 100 mg of the PSPs was suspended in water containing 7.5 mg L^−1^ of caffeine and stirred using a hot plate (300 rpm) and a magnetic stirrer bar (25 mm × 7 mm). The solution was kept at 25 °C during the experiments. At different time intervals, 2 mL aliquots were collected and filtered through a 0.22 μm filter for quantifying the residual concentrations of the caffeine by UV–Vis, using a Perkin Elmer Lambda 35 UV–vis spectrometer at 273 nm. All analyses were performed in duplicates. To evaluate the stabilities of caffeine (at 7.5 mg L^−1^) in water, the same procedure was followed, but without the addition of the PSPs, and no degradation was observed.

To better understand the adsorption mechanisms, thermodynamic studies were carried out at 35 °C and 45 °C, alongside studies at different pH and ionic strengths. The thermodynamic studies mirrored those at 25 °C, and control samples were employed at each temperature to confirm caffeine stability. The influence of pH on the adsorption of caffeine onto the PSPs was studied in the pH range of 1–10, maintaining the ionic strength constant at 0.5 mol L^−1^^14^. The concentration of caffeine and PSPs were 7.5 mg L^−1^ and 250 mg L^−1^, respectively. The experiments were prepared similarly to the adsorption kinetics assays, with the flasks being shaken during the previously determined apparent equilibration time (120 min), followed by quantification using UV–Vis. As previously reported by Diniz et al*.*^14^, caffeine is chemically stable under the entire range of pH studied. The influence of ionic strength was studied considering the range of NaCl concentration of 0.01–10 g L^−1^. The procedure was the same as used for the pH studies.

The adsorption isotherm studies were carried out using different amounts of PSP_40:60:1.000_ suspended in 400 mL of water containing the caffeine at a concentration of 7.5 mg L^−1^ and throughout the previously determined apparent equilibration time (120 min).

### Photocatalysis studies

Photocatalytic activity of all the PSPs was tested by using the Cole-Parmer Handheld UV Lamp of 6 W. For the photocatalytic degradation of caffeine under 254 nm, 25 mg of PSP was dispersed in 100 mL of caffeine solution (7.5 mg L^−1^). Then 500 μL of ethanol was added to the solution to reduce the surface tension and increase the dispersion of the PSPs^27^. The mixture was kept in the dark for the first 60 min, then irradiated and after several times (65, 70, 80, 90, 105, 120, 150 and 180 min) 2 mL portion of the mixture was sampled and filtrated 0.22 μm filter to separate the PSPs. The residual caffeine concentration was determined by UV–Vis.

## Results and discussion

### Characterization

**Figure 2:**
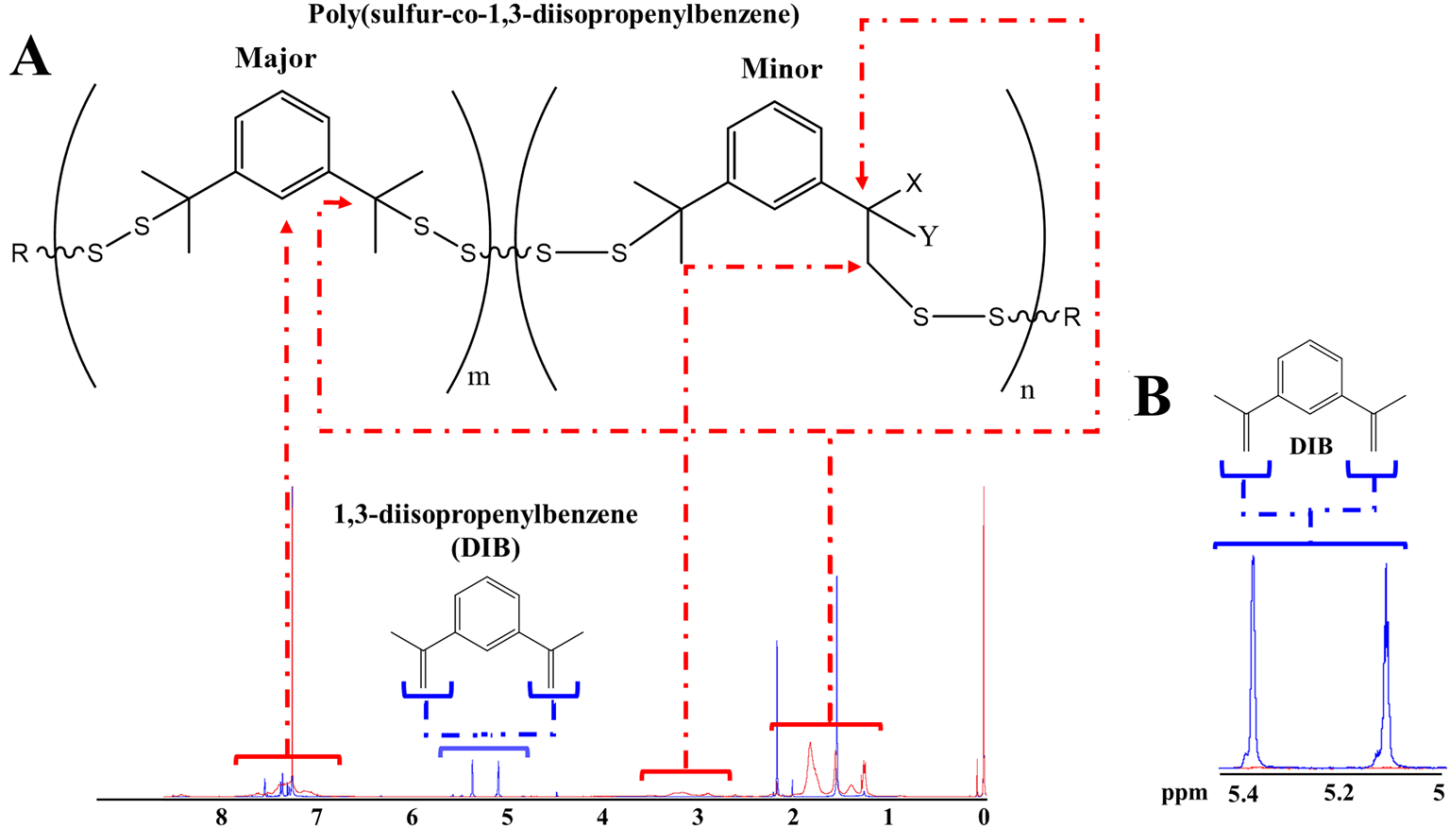
(**A**, **B**) Full and zoom-in of ^1^H-NMR spectrum of PSP_40:60:1.000_ (red) and 1,3-diisopropenylbenze (DIB) (blue) in CDCl_3_ after water treatment, respectively. R: -SH, Ph-C(CH_3_) = CH_2_; X: CH_3_, -Sn; Y: CH_3_, H. The poly(sulfur-co-1,3-diisopropenylbenzene) was adapted from Bao et al.^28^. PSP_X:Y:Z_ = X%[DIB]:Y%[S_8_]:Zg[NaCl].

Chung et al*.*^5^ first synthesized poly(sulfur-co-1,3-diisopropenylbenzene) copolymers in 2013. Their approach involved ring-opening polymerization at temperatures above 170 °C, converting S_8_ rings into sulfur diradicals chains. A cross-linking agent was slowly added at room temperature, leading to color change from yellow to red and increased viscosity. The main structure (Fig. 2A) of the copolymer poly(sulfur-co-1,3-diisopropenylbenzene) is predominantly composed of thiocumyl fragments, serving as the major building blocks. These are then accompanied by smaller segments consisting of either thiopropyl fragments or bis-thiopropyl fragments. Furthermore, the end groups of the poly(sulfur-co-1,3-diisopropenylbenzene) can be deduced to be either − SSH sulfanes or exposed isopropenyl moieties^28^.

**Figure 3:**
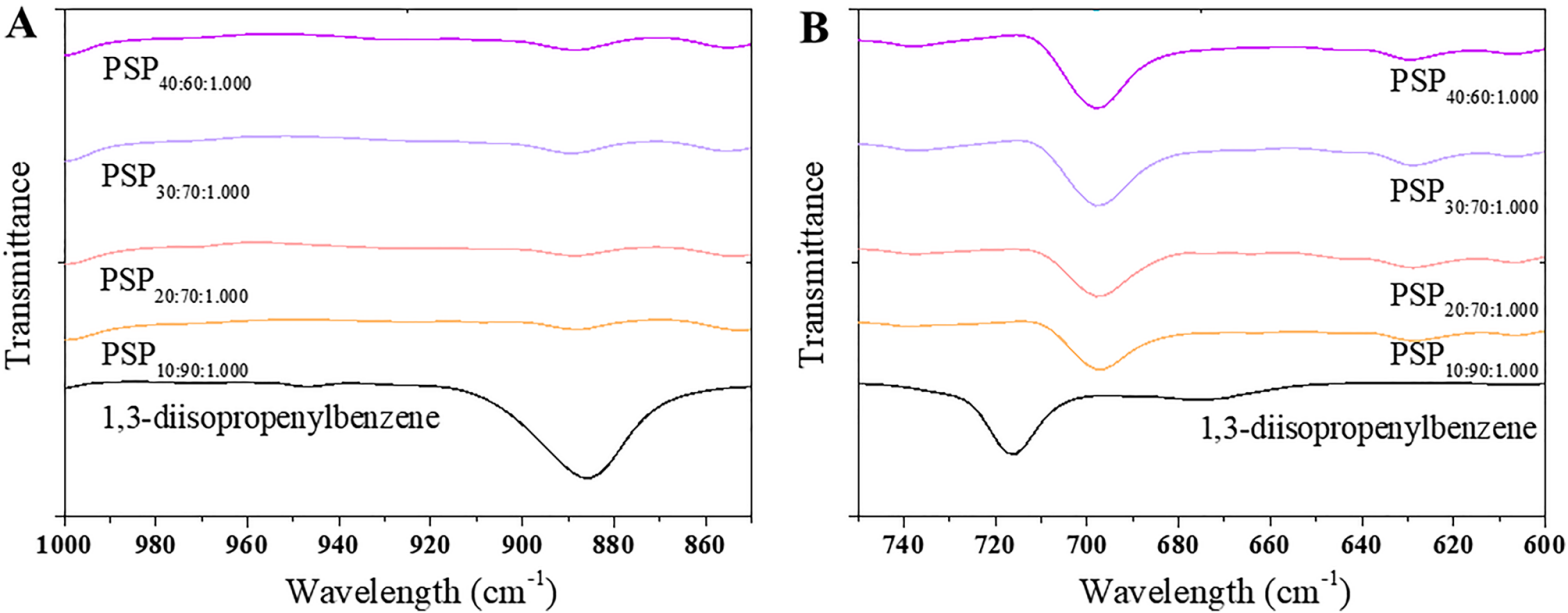
(**A**, **B**) FTIR spectra of the porous sulfur polymer (PSP) synthesized considering different sulfur/DIB ratios and 1.000 g of table salt after water treatment. PSP_X:Y:Z_ = X%[DIB]:Y%[S_8_]:Zg[NaCl].

Figure 2 and 3 display the ^1^H-NMR and FTIR (see Figure S3 for full range FTIR) spectra of PSP_40:60:1.000_. Due to overlapping with the deuterated solvent signal (CDCl_3_: proton signal at δ = 7.26 ppm), this spectrum can only be used qualitatively^29^. The extent of DIB consumption can be confirmed by the disappearance of methylene proton signals (δ = 5.10–5.40 ppm) in the PSP_40:60:1.000_ (Fig. 2B). This is also supported by the disappearance of the 900 cm^−1^ band in the FTIR spectra, indicating substantial consumption of double bonds during the crosslinking process^30^ (Fig. 3A). The detailed spectra of methyl protons at δ = 1.0–2.2 ppm indicated the formation of true copolymers through sulfur copolymerization (Fig. 2A). This complexity emerges due to the random polymerization, resulting in a final product with a heterogeneous composition consisting of polymers of varying sizes^5^. The peaks at δ = 6.80–7.80 ppm (Fig. 2A) are related to the presence of the aromatic rings of the DIB in the polymers^29^. Further, resonances between δ = 2.9–3.4 ppm (Fig. 2A), corresponding to methylene peaks in the PSP_40:60:1.000_ backbone, were observed. These peaks are linked to sulfur comonomer units, which is also supported by the appearance of a 692 cm^−1^ band in the FTIR spectra, indicating C-S bond formation (Fig. 3B)^31^. The PZC for the PSPs was also carried out (Figure S4), revealing an inherent correlation with the DIB content. Notably, an increase in DIB content corresponded to an elevated PZC value. For instance, PSP_10:90:1.000_ exhibited a PZC of 9.1, while PSP_40:60:1.000_ displayed a PZC of approximately 10.5.

The DSC curves offer insights into the sulfur conversion process and the glass transition of PSPs (Figure S5). Elemental sulfur exhibits a melting transition temperature of around 119 °C, attributed to the melting of monoclinic sulfur^6^. PSPs with over 10% wt. DIB showcase an absence of melting transition of sulfur, underscoring the amorphous nature of the co-polymers, which reinforces the structural characteristics observed in the NMR data. Furthermore, the glass transition temperature (T_g_) within the PSP series increased proportionally with elevated DIB content. Notably, PSP_20:80:1.000_ exhibits a minimum T_g_ of approximately − 5.4 °C, while PSP_40:60:1.000_ displays a maximum of about 14.9 °C. This T_g_ elevation stems from the higher DIB content leading to shorter sulfur–sulfur chain lengths in the copolymers. Consequently, chain mobility is constrained, resulting in the higher T_g_ values observed. The thermogram of PSP_10:90:1.000_ unveils residual monoclinic sulfur due to its elevated sulfur content relative to DIB. This excess sulfur diminishes the T_g_ value to − 17.6 °C. These findings align with those of Chung et al*.* (3), who observed similar trends during the synthesis of sulfur polymers through the “inverse vulcanization” process.

**Figure 4:**
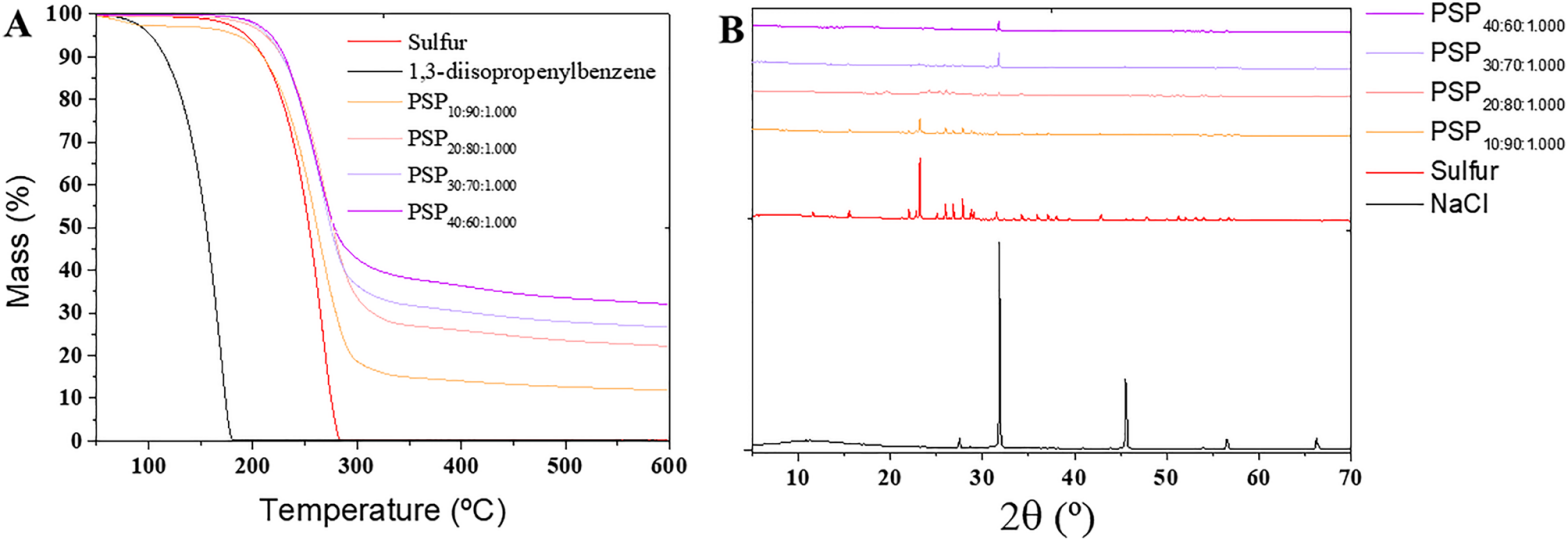
(**A**) Thermogravimetric thermograms and (**B**) Powder X-ray Diffraction spectra of the porous sulfur polymer (PSP) synthesized considering different sulfur/DIB ratios and 1.000 g of table salt after water treatment. PSP_X:Y:Z_ = X%[DIB]:Y%[S_8_]:Zg[NaCl].

The thermal stability assessment of PSPs was conducted via TGA. Figure 4A illustrates TGA thermograms for pure sulfur as well as PSPs with varying weight percentages of sulfur and DIB. The graph depicts that the degradation of pure sulfur commences at approximately 190 °C, with complete weight loss (100%) observed around 283 °C. However, the PSPs exhibited a higher decomposition temperature than pure sulfur. Additionally, the PSPs had a residue at 600 °C, and this residual content increased with a rise in DIB content within the composition.

PXRD patterns for pure sulfur, sodium chloride (NaCl), and PSPs after water treatment are presented in Fig. 4B. In the case of pure sulfur, characteristic diffraction peaks were discernible at 2θ = 23°, 27°, 28°, 53°, and 56°. However, these peaks were absent following the copolymerization reaction in polymers containing over 10% DIB content, which was already observed in the DSC thermograms. This absence suggests a transformation from crystalline monoclinic sulfur to a highly cross-linked amorphous copolymer structure. The diffraction peaks originating from pure table salt (NaCl at 2θ = 31°, 46°, 56°, and 66°) remained observable in PSPs after water treatment. This persistence indicates the presence of residual table salt even after the water washing treatment.

**Figure 5:**
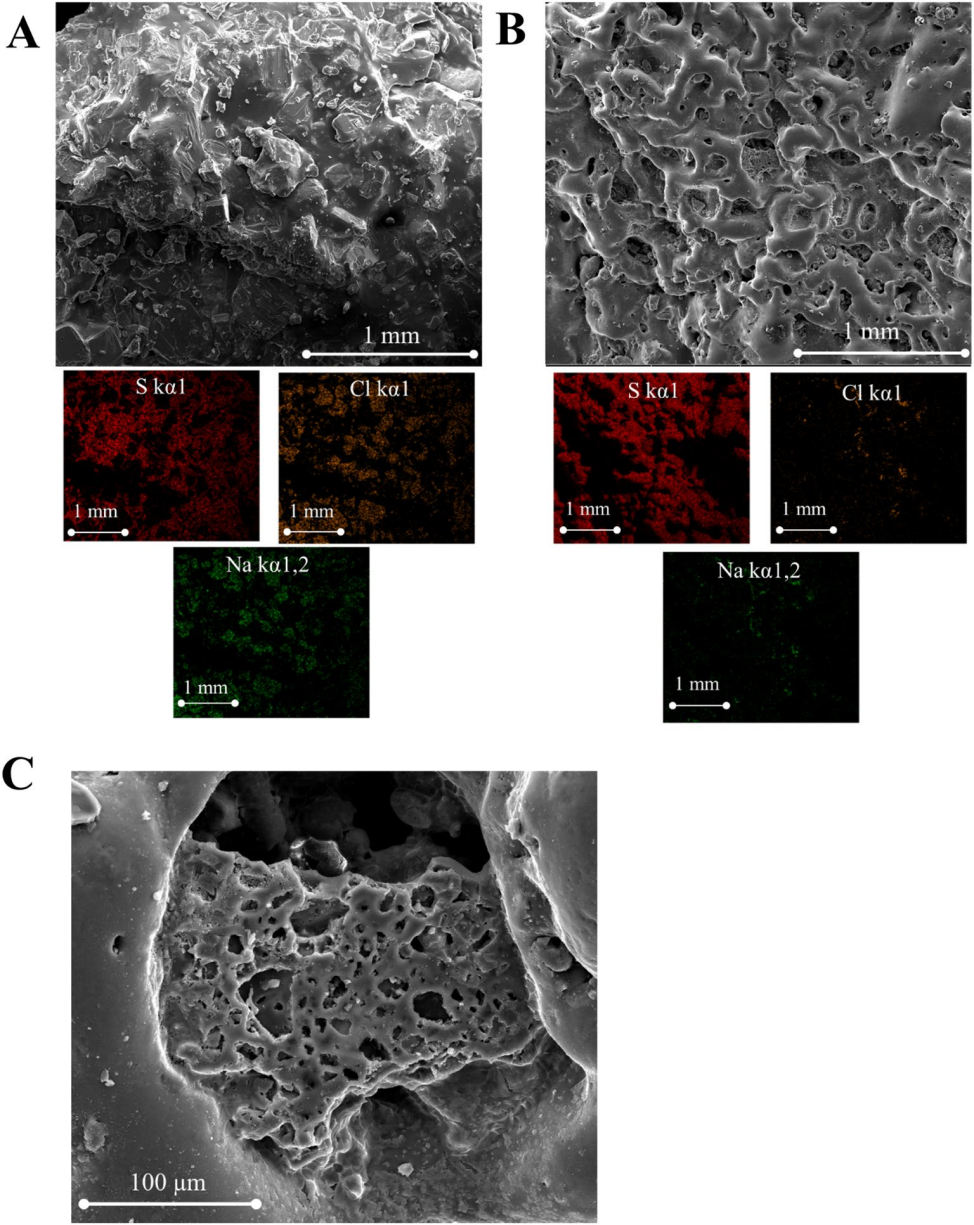
SEM image of PSP_40:60:1.000_ (**A**) before water treatment (insight image shows the Na, Cl, and S elemental mapping) and (**B**) after treatment with water (insight image shows the EDS of the sample). (**C**) High magnification image of the PSP_40:60:1.000_ after water treatment. PSP_X:Y:Z_ = X%[DIB]:Y%[S_8_]:Zg[NaCl].

SEM images of PSP_40:60:1.000_ before water treatment reveals a rugged and non-porous surface (Fig. 5A), comprising approximately 42.2% sulfur and 57.2% table salt (Table S1). Moreover, the SEM image indicates an even distribution of table salt and sulfur throughout the sample. Elemental mapping (specifically of Na, Cl, and S) provides validation that the crystal-like formations originate from the table salt surface. After water treatment, these salts were dissolved in water, prompting the development of a porous structure (Fig. 5B). The residual table salt content after treatment amounted to around 10.8% (Table S1), consistent with PXRD data. The non-uniform distribution of pore sizes can be ascribed to the irregular structure of NaCl crystals. Figure 5C illustrates the presence of pores of varying dimensions, adopting a mixed cellular configuration characterized by partially open and partially closed cell arrangements.

The link between the adsorption capacity of porous materials and their surface area is well-established^14,18,32–34^. Therefore, the surface area of the PSPs was examined using BET isotherms (Figure S6), and the corresponding data is detailed in Table S2. Notably, PSPs with a lower monomer content (PSP_10:90:1.000_) exhibited a higher surface area, primarily attributed to reduced volume shrinkage owing to a lower cross-linking density^6^. On the other hand, PSPs containing a higher proportion of DIB (PSP_40:60:1.000_) demonstrated lower surface area due to the heightened volume shrinkage linked with more pronounced cross-linking^6^.

To explore the impact of varying table salt quantities on the PSPs, PSP_40:60_ was synthesized using four different table salt amounts (0.250, 0.500, 1.000, and 1.500 g). The table salt was not observed to affect the structure of polymers, which can be seen in the FTIR spectra (Figure S7). In addition, no changes in the DSC curves were observed, with T_g_ remaining around 14.9 °C (Figure S8). On the other hand, the BET isotherm results revealed a proportional increase in surface area with higher table salt quantities, and an optimal table salt amount of 1.000 g was identified (Table S2). However, in the case of PSP_40:60:1.500_, a significant decrease in surface area was observed, attributable to the presence of a high content of residual table salt within the PSP even following water treatment. The higher residual of table salt can be confirmed by PXRD, TGA thermogram, and elemental mapping (Figures S8 and S9).

### Removal experiments

#### Adsorption studies

##### Kinetics studies and isotherm studies

The adsorption of caffeine onto the PSPs was first performed to estimate the apparent equilibrium time, the adsorption kinetics, and the adsorption potential. The apparent equilibrium time was around 120 min regardless of the PSP (Figure S10). For a more in-depth understanding of the kinetic characteristics of the adsorption process of caffeine by the PSPs, the pseudo-first order, pseudo-second order, Elovich, intraparticle diffusion, and film diffusion (see supplementary information for equations) models were used to fit the experimental data (Figure S9) and the relevant data parameters were listed in Table 1.

**Table 1:** Kinetics adsorption parameters of caffeine onto porous sulfur polymers (PSPs).

Model	Parameter	PSP_10:90:1.000_	PSP_20:80:1.000_	PSP_30:70:1.000_	PSP_40:60:1.000_	PSP_40:60:0.250_	PSP_40:60:0.500_	PSP_40:60:1.500_
Experimental	Q_ads_ (mg g^−1^)	9.81	10.56	13.50	21.73	7.33	13.20	9.45
Pseudo-first order	q_ads_ (mg g^−1^)	8.77	9.70	12.44	20.11	6.72	12.64	9.29
	K_1_ (min^−1^)	0.1521	0.0669	0.0575	0.0714	0.0683	0.0615	0.0573
	R^2^	0.937	0.955	0.953	0.963	0.974	0.965	0.983
	SD	0.754	0.740	0.978	1.411	0.414	0.866	0.444
Pseudo-second order	q_ads_ (mg g^−1^)	9.43	10.89	14.07	22.53	7.58	14.28	10.54
	K_2_ (g mg^−1^ min^−1^)	0.024	0.008	0.005	0.004	0.011	0.005	0.007
	R^2^	0.978	0.977	0.982	0.985	0.960	0.978	0.993
	SD	0.606	0.536	0.606	0.894	0.514	0.684	0.276
Elovich	α (mg ^g−1^ min^−1^)	27.20	2.62	2.51	5.83	1.50	2.74	1.758
	β (mg g^−1^)	0.848	0.502	0.368	0.245	0.690	0.367	0.484
	R^2^	0.994	0.974	0.982	0.976	0.913	0.965	0.977
	SD	0.234	0.562	0.609	1.128	0.760	0.862	0.519
Intraparticle diffusion	K_i1_ (mg g^−1^ min^−0,5^)	1.667	1.146	1.857	3.269	1.183	1.777	1.401
	K_i2_ (mg g^−1^ min^−0,5^)	0.354	0.455	0.960	1.113	0.428	0.442	0.659
	K_i3_ (mg g^−1^ min^−0,5^)	− 0.100	− 0.080	− 0.053	− 0.326	0.050	0.128	0.000
	C_i_	3.34	2.26	2.55	4.86	1.52	2.71	1.87
	R^2^	0.732	0.850	0.875	0.837	0.752	0.843	0.856
	SD	1.560	1.358	1.597	2.950	1.28	1.839	1.292
Film diffusion	K_fd_ (min^−1^)	0.348	0.030	0.037	0.039	0.039	0.037	0.046
	R^2^	0.526	0.615	0.911	0.950	0.844	0.972	0.985
	SD	0.577	0.510	0.217	0.249	0.306	0.240	0.181

PSP_X:Y:Z_ = X%[DIB]:Y%[S_8_]:Zg[NaCl].

The fitting coefficient of the pseudo-second-order model (R^2^) was larger than that of the pseudo-first-order model, and the standard deviation (SD) of the pseudo-second-order model was lower than the pseudo-first-order model. Additionally, the theoretical adsorption capacity (q_ads_) values calculated by the pseudo-second-order model were also closer to the experimental values (Q_ads_) (Table 1). The pseudo-second-order model is better suited to describe the adsorption kinetics of caffeine onto the PSP, which also suggests that the adsorption process was governed by chemical adsorption^35^. The good fitting coefficients of the Elovich model are also an indicator of a chemisorption process^14,36,37^. Furthermore, achieving a good fit to the Elovich model holds significant recognition as evidence of sorption site heterogeneity^38^. This heterogeneity denotes the presence of diverse surface types, including micro- and mesopores, which in turn engender multiple sorption steps. These steps encompass phenomena like "monolayer–multilayer" adsorption, capillary condensation, and the filling of pores of varying sizes^39^.

The process of adsorption is commonly delineated into three consecutive stages: (I) external mass transport, (II) film diffusion, and (III) intraparticle diffusion. In batch studies, the initial stage is often overlooked due to the vigorous agitation employed^14^. Proficiency in numerically characterizing the second and third stages is demonstrated by the film and intraparticle diffusion models, respectively. These models not only provide insights into the rate-controlling phases of adsorption^40^ but also uncover multi-linear adsorption kinetics. Importantly, it was notable that the fit of the intraparticle diffusion model does not originate from the origin, signifying the participation of two or more steps in constraining the adsorption process^14,41^.

The findings suggest the segmentation of the adsorption process into several stages, aligning with observations made for other contaminants^40,42^. The intraparticle’s diffusion rate progressively diminished across these stages (K_i1_ > K_i2_ > K_i3_) (Table 1), underscoring the influence of pore diffusion on the overall adsorption pace. In addition, as the film diffusion constants (K_fd_) were lower than the corresponding intraparticle’s constant (C) values (Table 1), film diffusion had a lower impact on the adsorption of caffeine onto PSPs than pore diffusion.

**Figure 6:**
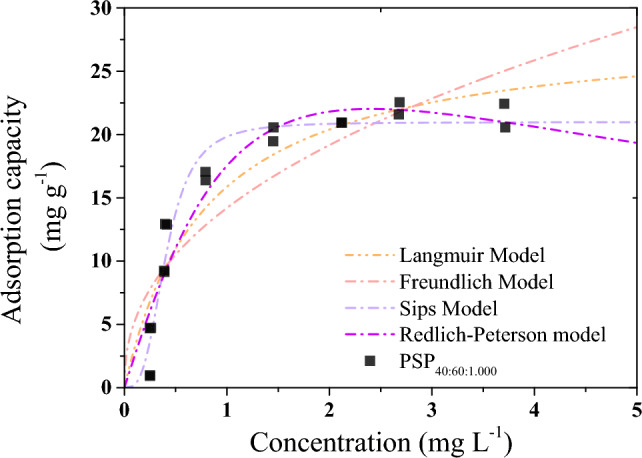
Effect of initial PSP_40:60:1.000_ loading on the adsorption of caffeine. PSP_X:Y:Z_ = X%[DIB]:Y%[S_8_]:Zg[NaCl].

To gain a more comprehensive comprehension of the interactions between the adsorbent and the adsorbate, equilibrium isotherms were evaluated for PSP_40:60:1.000_, leveraging the previously established apparent equilibrium time of 120 min. Four distinct isotherm models (see supplementary information for equations) were investigated: Langmuir, Freundlich, Redlich-Peterson, and Sips. The isotherms exhibited a type L2 shape^43^ (Fig. 6), signifying a concave curvature, which indicates a favorable adsorption scenario^43^. This curvature suggests that as active sites on the surface of the adsorbent material became occupied, the likelihood of the compound encountering an available active site diminished, further substantiating the notion of adsorption favorability.

The Sips model provided the best fitting for the adsorption of caffeine onto PSP_40:60:1.000_ since R^2^ and SD values were higher and lower, respectively, than the values obtained by the other three models. In this context, at low adsorbate concentrations, the Sips model takes on the characteristics of the Freundlich isotherm. However, as the adsorbate concentration increases, it transitions into the Langmuir isotherm. This phenomenon elucidates the distribution of adsorption energies across the heterogeneous surface of the adsorbent^44^. Therefore, the Sips combines the multilayer adsorption of the Freundlich model with the monolayer adsorption of the Langmuir model, evidencing a "monolayer–multilayer” adsorption onto a heterogeneous surface of the adsorbent. The isotherm results agree with the good fitting of the Elovich model already observed. In addition, the q_ads_ value (20.97 mg g^−1^) obtained by the Sips models was very similar to the experimental one (Table 2).

**Table 2:** Equilibrium adsorption parameters of caffeine onto PSP_40:60:1.000Tab_.

Model	Parameter	PSP_40:60:1.000_	Model	Parameter	PSP_40:60:1.000_
Langmuir	K_L_ (L mg^−1^)	1.25	Redlich-Peterson	K_RP_ (L mg^−1^)	25.43
	q_ads_ (mg g^−1^)	28.54		$\alpha_{RP}$((mg g^−1^)^g^)	0.45
	R^2^	0.871		g	1.56
	SD	2.83		R^2^	0.908
				SD	2.47
Freundlich	K_F_ ((mg/g)/(mg/L)^n^)	14.2	Sips	Ks (L mg^−1^)	2.46
	n	2.31		q_ads_ (mg g^−1^)	20.97
	R^2^	0.779		n	3.17
	SD	3.7		R^2^	0.955
				SD	1.72

PSP_X:Y:Z_ = X%[DIB]:Y%[S_8_]:Zg[NaCl].

Activated carbon is a porous material well-known for removing organic contaminants and small molecules from aqueous solution due to its high surface area, well-developed porosity, and high adsorption capacity^18,45^. In this work, the adsorption of caffeine by a commercially available activated carbon was performed and compared to the results from the adsorption of caffeine onto the PSPs. The q_ads_ values were normalized by the surface area of the adsorbent (Eq. 1). The results indicated that the adsorption per square meter (m^2^) onto the PSPs was at least 2.1 times higher when compared to the activated carbon (Table S3). This observation underscores the more efficient nature of the adsorption process facilitated by the PSPs. Furthermore, when evaluating the results in the context of similar studies available in scientific literature, the superiority of PSPs remains evident even when confronted with higher initial concentrations of caffeine that inherently favor the adsorption process (Table S3).

1 $${q}_{norm}= \frac{{Q}_{ads}}{SA}$$

where q_norm_ is the normalized adsorption capacity (mg m^−2^) and SA is the surface area (m^2^ g^−1^).

It is also important to note that although increasing the DIB content leads to materials with lower surface area, the normalized adsorption increases. This observation indicates that the monomer plays a crucial role in the adsorption of caffeine (Table S3). Adsorption experiments using another emerging contaminant (saccharin) with the PSPs synthesized with different content of monomers (PSP_10:90:1.000_, PSP_20:80:1.000,_ PSP_30:70:1.000,_ PSP_40:60:1.000_) were also carried out to confirm the effects of DIB on the adsorption properties of the material. The results also showed that the higher the content of the DIB, the higher the normalized adsorption capacity (Figure S11). To further understand the mechanisms involved in this process, adsorption studies at different pH, ionic strength, and temperature were carried out using the PSP that provided the best adsorption results (i.e., PSP_40:60:1.000_).

#### Adsorption mechanism

**Figure 7:**
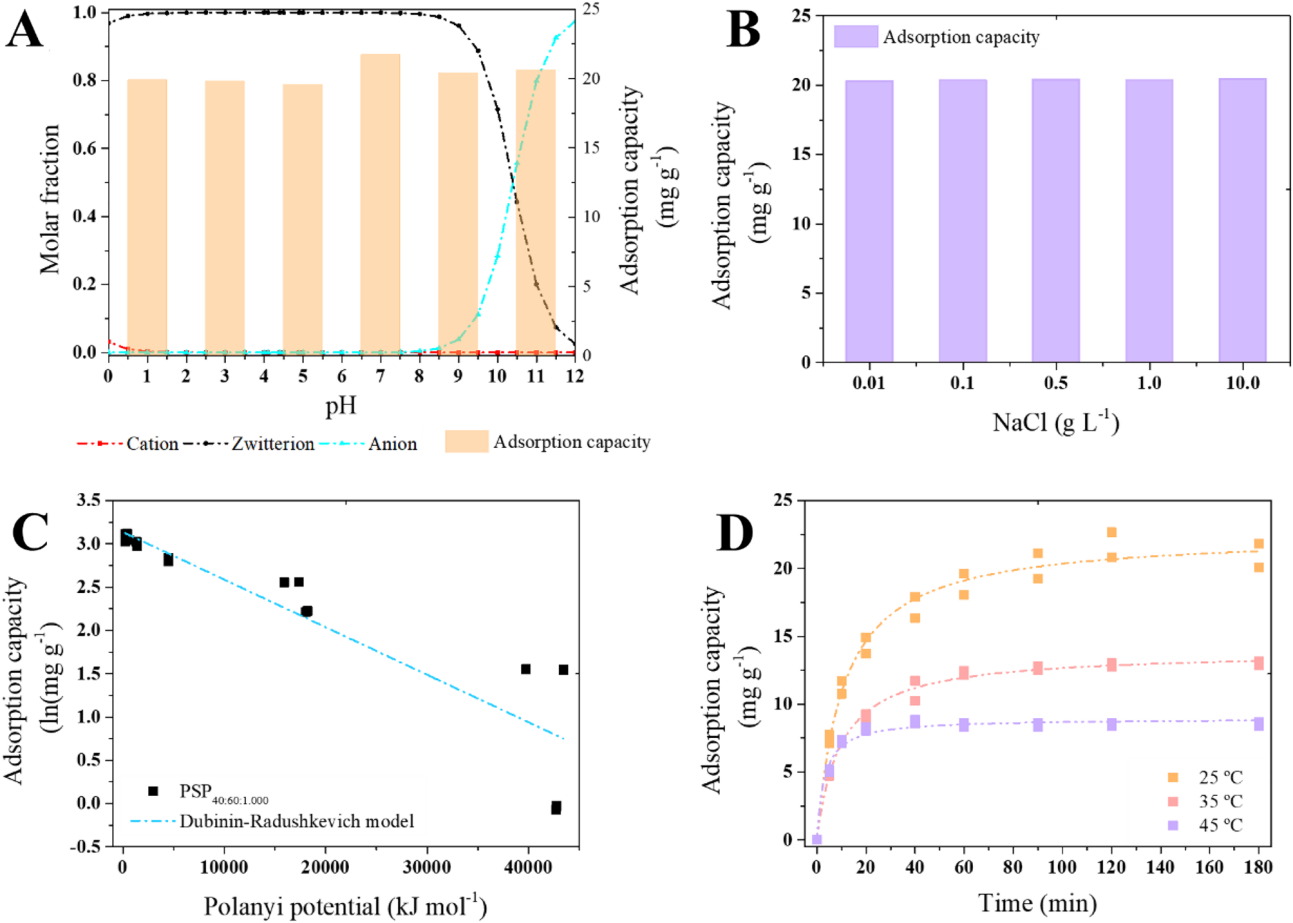
(**A**) Influence of pH, (**B**) ionic strength, (**C**) Fitting to the Dubinin-Radushkevich model, and (**D**) temperature in caffeine (CAF) adsorption onto PSP_40:60:1.000_, and. PSP_X:Y:Z_ = X%[DIB]:Y%[S_8_]:Zg[NaCl].

One of the key variables in the adsorption process is the pH of the medium, which in turn modulates the surface charge and functional groups of the adsorbent and the charge speciation of the chemical compounds^18,46^. Caffeine occurs predominantly in zwitterion form at pH below 9 and in negative form at pH higher than 11 (Fig. 7A). According to the adsorption results, the q_ads_ values were similar in the range of pH studied, which indicates that electrostatic forces did not govern the adsorption of caffeine onto PSP_40:60:1.000_^47^, which was further confirmed by the studies carried at different ionic strengths (Fig. 7B).

The Dubinin-Radushkevich model exhibited a R^2^ value of 0.845 (Fig. 7C). This model may provide interesting insights into the adsorption of caffeine onto PSP_40:60:1.000_. The model constant value (K_DR_) enables the estimation of the mean adsorption energy (E), which is defined as the free energy transfer of 1 mol of solute from infinity to the surface of the adsorbent^48^. The magnitude of the E value offers insight into the adsorption mechanism: E values ranging from 4 to 8 kJ mol^−1^ imply weak bonds such as van der Waals interactions or physisorption, E values from 2 to 40 kJ mol^−1^ signify hydrogen bonding, and E values above 60 kJ mol^−1^ correspond to chemisorption involving valence bond forces of chemical bonds^49–51^.

In the case of the caffeine adsorption onto PSP_40:60:1.000_, the calculated E value was 95.40 kJ mol^−1^, suggesting the prevalence of chemisorption. This outcome aligns with expectations, considering the adsorption was unaffected by pH and ionic strength (Fig. 7). Additionally, the q_ads_ value of the Dubinin-Radushkevich model was 23.00 mg g^−1^, which was similar to the experimental result (Table 2).

However, to confirm whether or not the adsorption of caffeine onto PSP_40:60:1.000_ belongs to chemical adsorption or physical adsorption, the activation energy (E_a_) of the reaction was also explored by kinetics thermodynamic studies (Fig. 7D)^52^. The adsorption E_a_ of caffeine onto PSP_40:60:1.000_ was determined using the PSO rate constant (K_2_) at different temperatures (Fig. 7D) and the Arrhenius equation, as previously described by Wang et al*.*^35^.

The fitting results of the experimental data are shown in Figure S12. The calculated E_a_ (*i.e.*, 87.2 kJ mol^−1^) also confirmed that the adsorption of caffeine onto PSP_40:60:1.000_ is an activated chemical adsorption process. The value was similar to the adsorption energy obtained by Zanella et al.^53^ in the adsorption of caffeine by activated biochar derived from macrophytes. Therefore, the adsorption of caffeine onto PSP_40:60:1.000_ belongs to a "monolayer–multilayer" chemisorption as already predicted by the good fitting on pseudo-second order, Elovich, and Sips models.

In addition to the determination of E_a_ values, kinetics thermodynamic studies can be used to calculate the standard enthalpy (ΔH^#^), and entropy of activation (ΔS^#^) by the Eyring equation. The free energy of activation (ΔG^#^) was also obtained by the ΔH^#^ and ΔS^#^ values.

The fitting of the experimental data (Figure S13) resulted in a $\Delta {H}^{\#}$ of 83.7 kJ mol^−1^ and $\Delta {S}^{\#}$ of – 43.15 J mol^−1^. These values are indicative of an exothermic process with an associative mechanism, which means that adsorbate does not cleave bonds but forms a complex compound during the chemisorption process^48,54^. The large positive values of $\Delta {G}^{\#}$ (70.8, 70.4, and 70.0 kJ mol^−1^) values indicate that energy (e.g., agitation) was required in the adsorption reaction to convert reactants into products^48^.

The adsorption of organic contaminants such as caffeine usually occurs by combining several mechanisms^14,55^. However, it has been reported that hydrophobic interactions significantly contribute to the adsorption of caffeine onto porous materials since the highest q_ads_ values are frequently reported for neutral caffeine species^14^. In the present study, it was observed that increasing the content of DIB, which also increased the content of benzene rings in the PSPs, led to higher adsorption of caffeine. Caffeine can form complexes with benzene rings due to aromatic molecule associations mainly in the 7-nitrogen region, acting as an electron acceptor in these complexes^56^. In addition, the amide group of caffeine molecule can form NH/π geometries of face-on type with benzene rings with energies higher than 40 kJ mol^−1^^57^ and therefore benzene rings can interact strongly with amide groups wing to their higher π–π stack bonding energies and hydrogen bonding^58,59^. However, due to the high free adsorption energy, it is expected that caffeine molecules had integrated into the PSPs structure, as Dittmann et al*.*^60^ observed in their studies with carbamazepine and activated carbons. Nevertheless, further studies should be conducted to investigate the adsorption mechanisms of caffeine and other organic pollutants into PSPs and also the effects of using other monomers on the adsorption properties.

### Photocatalysis studies

**Figure 8:**
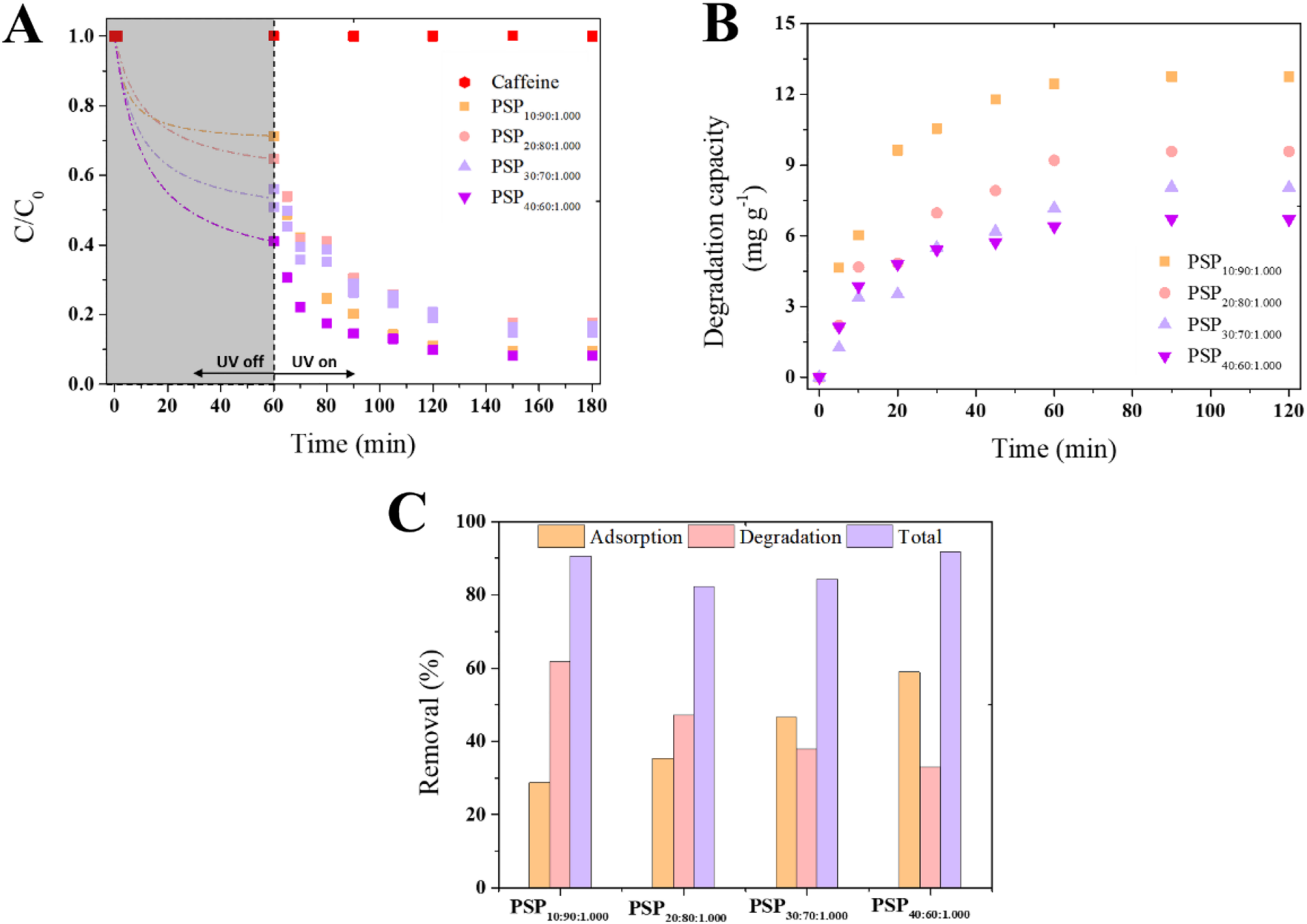
(**A**) Removal of caffeine by porous sulfur polymers (PSPs) considering 60 min of adsorption and 120 min of irradiation (254 nm). The dash-dot lines indicate the simulated pseudo-second order adsorption process. (**B**) Normalized degradation capacity of the PSPs. (**C**) Estimation of the contribution of adsorption and degradation to caffeine removal by PSPs. PSP_X:Y:Z_ = X%[DIB]:Y%[S_8_]:Zg[NaCl].

The light absorbance of the PSPs was investigated using UV–Vis spectroscopy (Figure S14). All PSPs exhibited an extended light absorption range compared to pure S_8_ and DIB. To be specific, the PSPs absorbed light across a broad spectrum, ranging from visible (475 nm) to UV (250 nm) regions. In contrast, pure S_8_ absorbed light from 350 nm and downwards. Therefore, the photocatalytic properties of the PSPs were tested for the degradation of caffeine. For equilibrium to be reached, the solutions were left in the dark for 60 min, during which the PSPs with higher DIB content exhibited greater adsorption capabilities (Fig. 8A), as expected. Conversely, the higher sulfur-content polymers showed higher photocatalytic activity (Fig. 8B). PSP_10:90:1.000_ showcased a degradation capacity of 12.8 mg g^−1^, which was 1.9 times higher than PSP_40:60:1.000_.

While previous research has proven the photocatalytic activity of sulfur by doping metal and non-metal^61,62^, and some studies also showed that high-sulfur content polymers have a potential photocatalytic activity^8,27,63^, this study pioneers the investigation of the ability of the PSPs to remove emerging contaminants, such caffeine, through a dual-action mechanism (adsorption and photocatalysis). The PSPs achieved up to 92% of caffeine removal when both adsorption and photocatalysis were combined. Whereas almost no adsorption occurs after 60 min (< 10%) (Figure S10), it can be observed that the removal of caffeine by higher sulfur content PSPs was governed by the photocatalytic activity, while PSPs with higher DIB content leaned towards the adsorption process (Fig. 8C). As an illustration, in the case of PSP_10:90:1.000_, approximately 28.7% of the caffeine removal can be attributed to the adsorption mechanism, while 61.8% is ascribed to the photocatalysis activity. Conversely, in the case of PSP_40:60:1.000_, the adsorption mechanism accounts for 58.9% of caffeine removal, whereas photocatalysis is responsible for 32.8% of caffeine removal. As a first report, this exciting achievement opens a new field of investigation, and further studies can explore modifications on the polymers and the removal of other emerging contaminants.

## Conclusions

The present study reports the synthesis of PSPs by “inverse vulcanization” using DIB as monomers and introducing porosity via salt templating that can remove caffeine by dual-action mechanism (adsorption and photocatalysis). The adsorption studies revealed a strong correlation between DIB content in the PSPs and their adsorption capacity, with higher DIB content PSPs displaying greater adsorption abilities. The thermodynamic analysis yielded E_a_ values of 87.2 kJ mol^−1^, ΔH# of 83.7 kJ mol^−1^, and ΔS# of – 43.15 J mol^−1^, indicating an exothermic chemisorption process that results in the formation of complexes between the benzene ring of the polymers and 7-nitrogen region of caffeine. The PSPs also showed extended light absorption spectra ranging from 475 nm into the UV region. This broad spectrum enabled photocatalytic activity that correlated with sulfur content in the PSPs, with higher sulfur content PSPs demonstrating superior photocatalytic potential. Notably, all the PSPs showed a dual-action mechanism that resulted in the removal of caffeine by up to 92%. However, it was demonstrated that for PSPs with higher sulfur content, the removal was governed by a degradation mechanism. Meanwhile, for those with higher DIB content, adsorption was the primary removal mechanism. The present study highlights the potential of utilizing PSPs to address the issue of sulfur waste responsibly, showcasing their capacity to generate value-added products through the dual-action mechanism of removing organic contaminants. The versatile synthesis of sulfur polymers offers variation, which means that there is much more to explore in this exciting research area.

### Supplementary Information

Supplementary Information.

## Data Availability

The datasets used and analysed during the current study are available from the corresponding author upon reasonable request.
