# Supplementary material for: Porous sulfur polymers for effective aqueous-phase organic contaminant removal
Source: Sci Rep. 2024 Apr 7;14:8144. doi: 10.1038/s41598-024-57856-8 (PMC10999450; doi:10.1038/s41598-024-57856-8)
Supplement: Supplementary file 1 — Supplementary Information. [file 41598_2024_57856_MOESM1_ESM.docx]

**Supplementary Information: Porous sulfur polymers for effective aqueous-phase organic contaminant removal**

Vinicius Diniz^1,2^, Joseph C. Bear^3^, Susanne Rath^2^ and Colin R. Crick^1*^

^1^School of Engineering and Materials Sciences, Queen Mary University of London, London, E1 4NS, UK

^2^Institute of Chemistry, University of Campinas, 13083-970 Campinas, Brazil

^3^School of Life Sciences, Pharmacy and Chemistry, Kingston University, Penrhyn Road, Kingston-upon-Thames, KT1 2EE, UK

*Author for correspondence: *c.crick@qmul.ac.uk*

**LIST OF FIGURES**

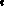

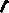


**Figure S1:** Global freshwater withdrawals for agricultural, industrial, and domestic uses by aggregated regional groupings (left side). Renewable internal freshwater resources flows refer to internal renewable resources (internal river flows and groundwater from rainfall) (right side). OECD members are defined as countries who were members in 2010 and their membership was carried back in time. BRICS countries are Brazil, Russia, India, China and South Africa. ROW refers to the Rest of the World, excluding OECD and BRICS countries. Data source Our world in data ^1^.

1. Our World in Data, <https://ourworldindata.org/>, (accessed 07th of August, 2023).

**Figure S2:** Approximate cost for (A) each treatment process considering a production of 100 m^3^/day of potable water and (B) considering a convention approach (activated sludge + coagulation & flocculation), general direct potable reuse (DPR, reverse osmosis + mineralization + activated carbon), and general DPR + membrane bioreactor (MBR). The calculations were based on the work of Guo et al.^1^

1. T. J. Guo, J. Englehardt and T. T. Wu, *Water Sci Technol*, 2014, **69**, 223-234.

**Figure S3:** Fourier Transform Infrared (FTIR) spectra of the 1,3-diisopropenylbenzene (DIB) monomer and porous sulfur polymer (PSP) synthesized considering different sulfur/DIB ratios and 1.000 g of table salt after water treatment. PSP_X:Y:Z_ = X%[DIB]:Y%[S_8_]:Zg[NaCl].


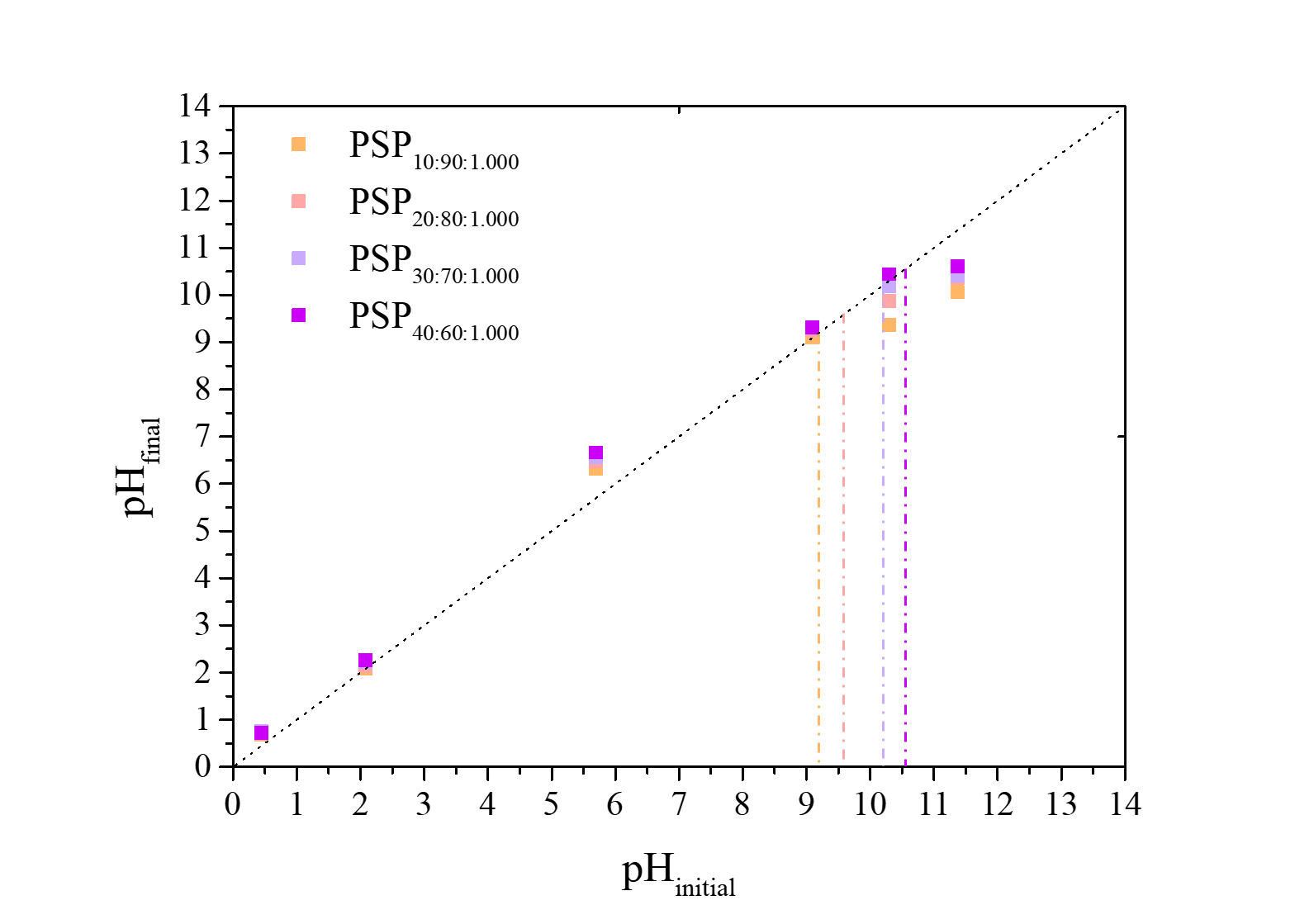


**Figure S4:** Point of zero charge of the porous sulfur polymers (PSPs) synthesized considering different sulfur/1,3-diisopropenylbenzene (DIB) monomer ratios. PSP_X:Y:Z_ = X%[DIB]:Y%[S_8_]:Zg[NaCl].

**Figure S5:** Differential Scanning Calorimetry (DSC) thermograms of 2^nd^ cycles of (A) PSP_10:90:1.000_, (B) PSP_20:80:1.000_, (C) PSP_30:70:1.000_, (D) PSP_40:60:1.000_, (E) Pure sulfur. PSP_X:Y:Z_ = X%[DIB]:Y%[S_8_]:Zg[NaCl].

**Figure S6:** Nitrogen adsorption isotherms of the porous sulfur polymers (PSPs). PSP_X:Y:Z_ = X%[DIB]:Y%[S_8_]:Zg[NaCl].


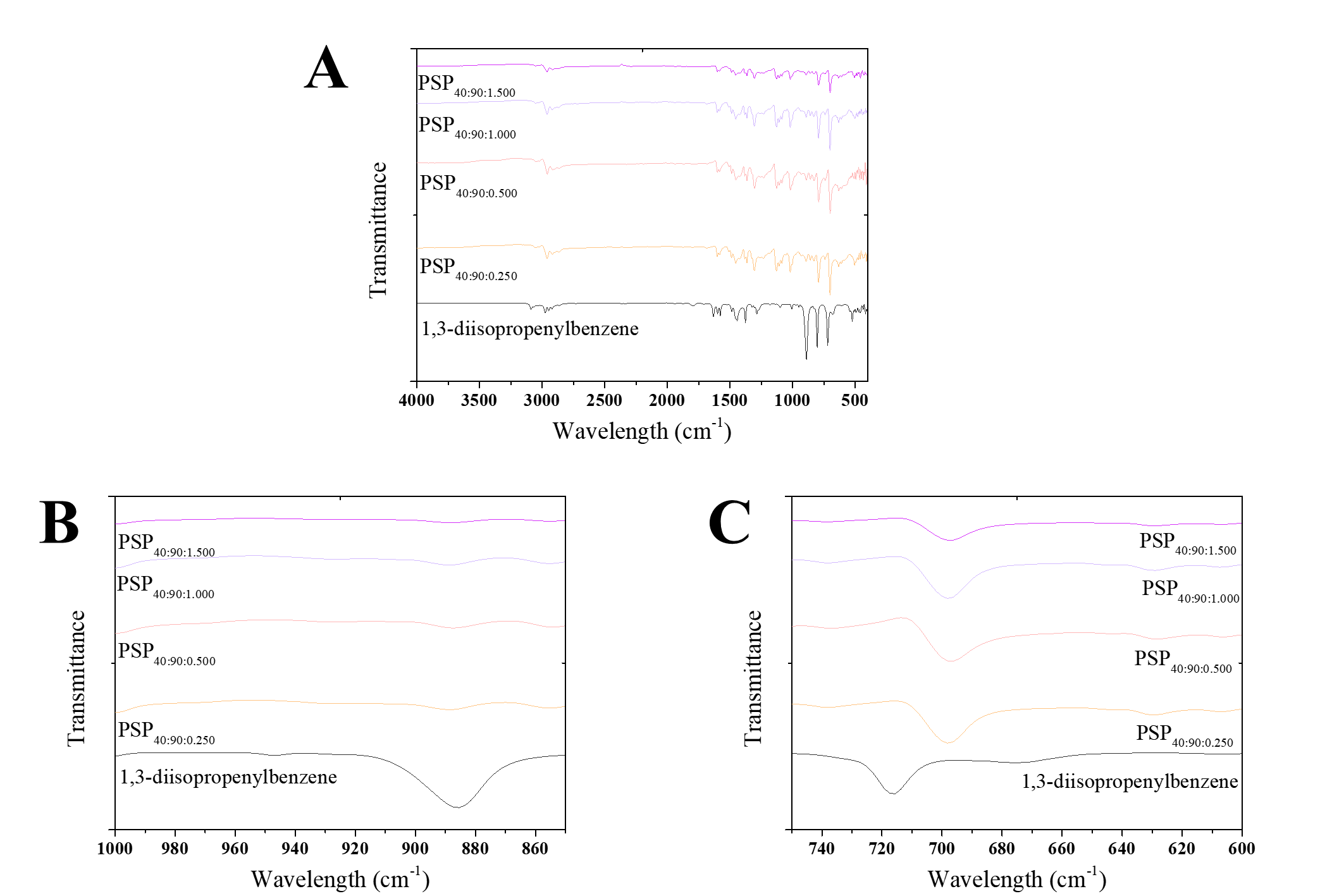


**Figure S7:** Fourier Transform Infrared (FTIR) spectra of the 1,3-diisopropenylbenzene (DIB) monomer and porous sulfur polymer (PSP) synthesized different amounts of salt. PSP_X:Y:Z_ = X%[DIB]:Y%[S_8_]:Zg[NaCl].


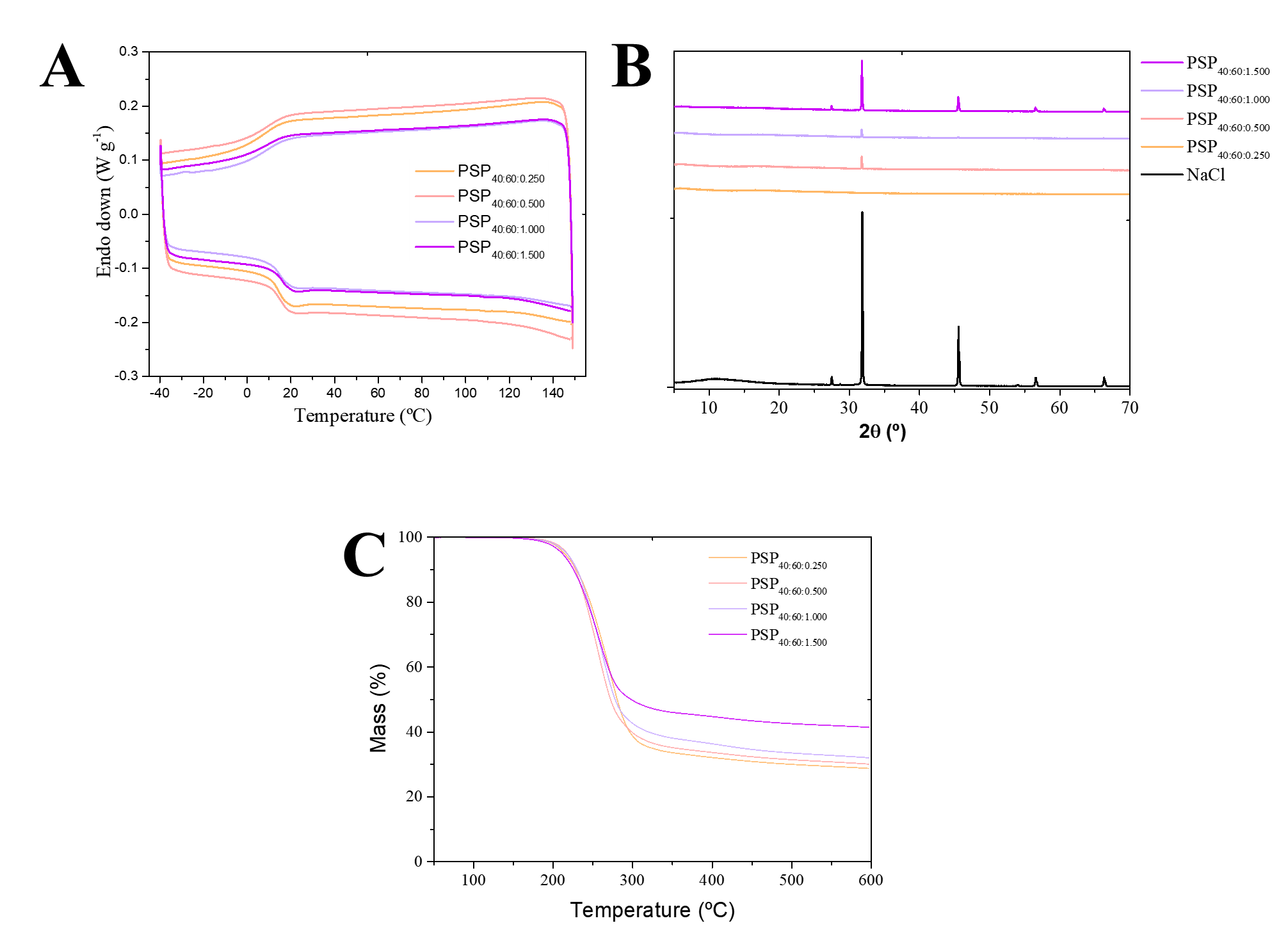


**Figure S8:** (A) Powder X-ray Diffraction, (B) Thermogravimetric analysis thermograms, and (C) Differential Scanning Calorimetry thermograms of the 1,3-diisopropenylbenze monomer and porous sulfur polymers (PSPs) synthesized considering different amounts of salt. PSP_X:Y:Z_ = X%[DIB]:Y%[S_8_]:Zg[NaCl].


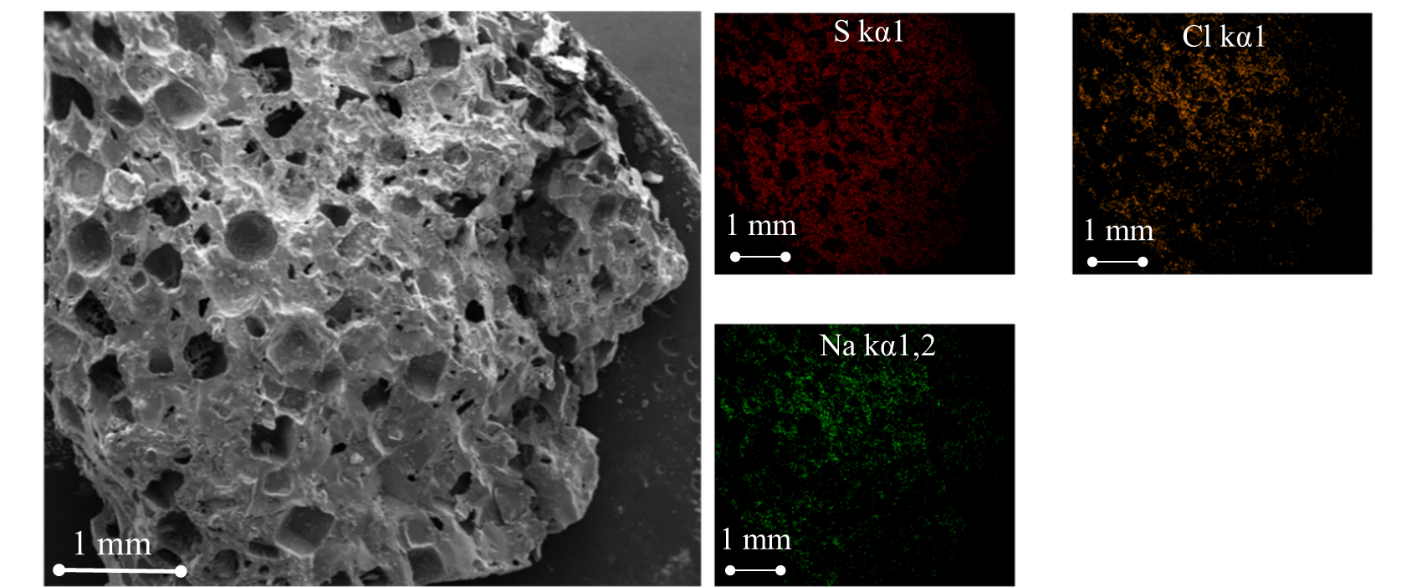


**Figure S9:** Scanning Electron Microscopy micrographs of PSP_40:60:1.500_ after water treatment (insight image shows the Na, Cl, and S elemental mapping). PSP_X:Y:Z_ = X%[DIB]:Y%[S_8_]:Zg[NaCl].


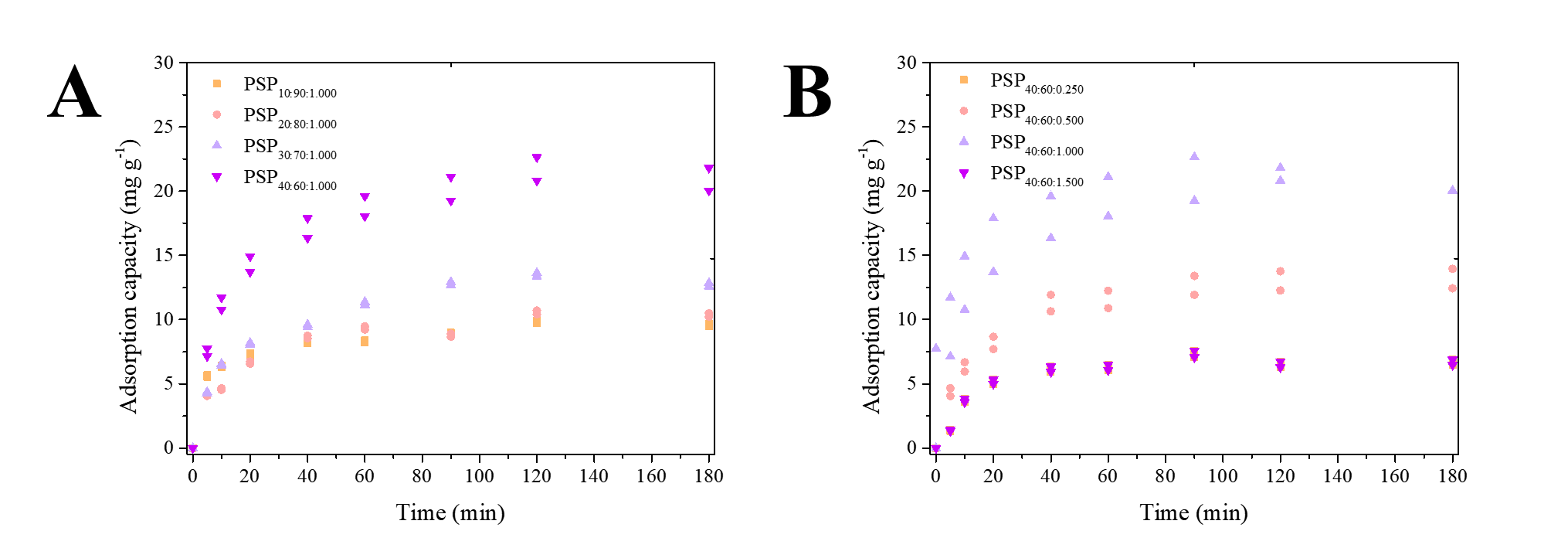


**Figure S10:** Adsorption kinetics of caffeine onto the porous sulfur polymers (PSPs). PSP_X:Y:Z_ = X%[DIB]:Y%[S_8_]:Zg[NaCl]. Initial concentration of caffeine 7.5 mg/L and PSP loading 250 mg/L

**Figure S11:** Normalized adsorption capacity of saccharin onto activated carbon and porous sulfur polymers. PSP_X:Y:Z_ = X%[DIB]:Y%[S_8_]:Zg[NaCl]. Initial concentration of saccharin 7.5 mg/L and PSP loading 250 mg/L


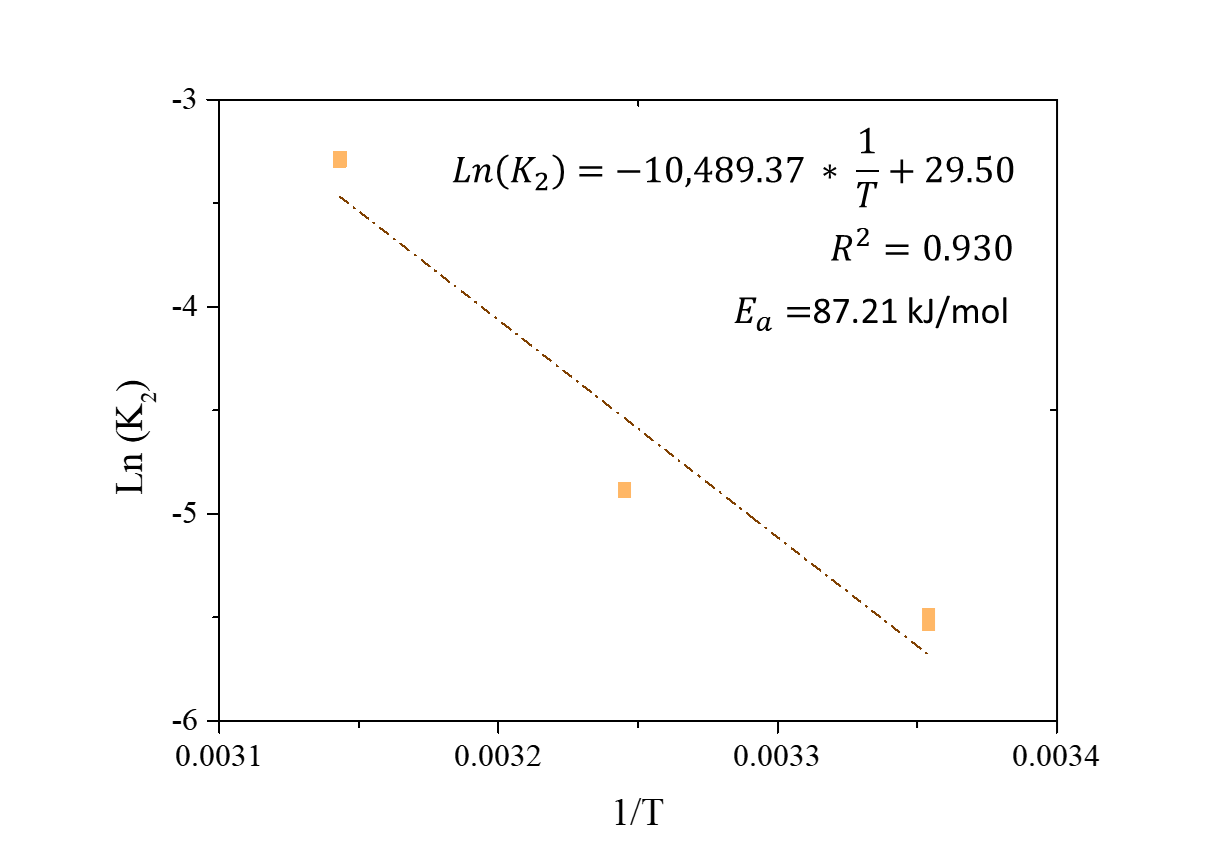


**Figure S12:** Influence of temperature on thermodynamic adsorption of caffeine and Arrhenius equation plot for adsorption of caffeine on PSP_40:60:1.000._ PSP_X:Y:Z_ = X%[DIB]:Y%[S_8_]:Zg[NaCl].


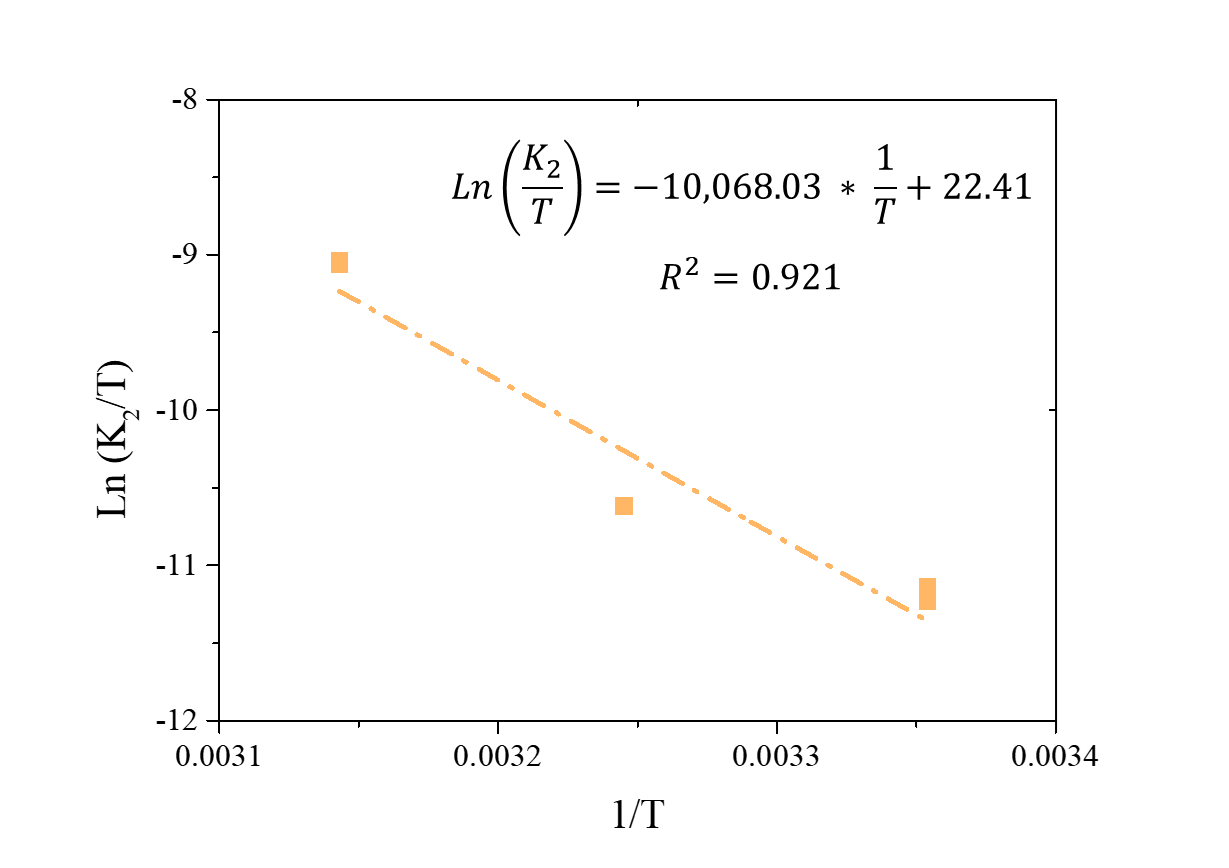


**Figure S13:** Influence of temperature on thermodynamic adsorption of caffeine and Eyring equation plot for adsorption of caffeine on PSP_40:60:1.000._ PSP_X:Y:Z_ = X%[DIB]:Y%[S_8_]:Zg[NaCl].

**Figure S14:** Ultraviolet-visible spectra of porous sulfur polymers (PSPs) from 900–250 nm, 1,3-diisopropenylbenze (DIB), and sulfur dissolved in chloroform (5 ng/mL)**.** The inset graph shows a zoom of the 500-350 nm region of the spectra. PSP_X:Y:Z_ = X%[DIB]:Y%[S_8_]:Zg[NaCl].

**LIST OF TABLES**

**Table S1:** EDS spectra values of the PSP_40:60:1.000_ before and after water treatment, respectively.

| **PSP_40:60:1.000_** | **Sulfur (%)** | **Chlorine (%)** | **Sodium (%)** |
| --- | --- | --- | --- |
| Before water treatment | 42.2 (0.3) | 35.9 (0.3) | 21.9 (0.3) |
| After water treatment | 89.2 (0.3) | 6.8 (0.2) | 3.9 (0.2) |

PSP_X:Y:Z_ = X%[DIB]:Y%[S_8_]:Zg[NaCl].

**Table S2:** Surface area of the porous sulfur polymers.

| **1,3-Diisopropenylbenzene (%)** | **Sulfur (%)** | **Table salt (g)** | | **Surface area**  **(m^2^ g^-1^)** |
| --- | --- | --- | --- | --- |
| 10 | 90 | 1.000 | 69.55 | |
| 20 | 80 | 1.000 | 69.21 | |
| 30 | 70 | 1.000 | 64.30 | |
| 40 | 60 | 1.000 | 61.30 | |
| 40 | 60 | 0.250 | 22.03 | |
| 40 | 60 | 0.500 | 52.43 | |
| 40 | 60 | 1.500 | 29.30 | |

**Table S3:** Comparison of adsorption capacity of different adsorbents for caffeine.

| **Material** | **Initial Concentration (mg L^-1^)** | **Normalized adsorption capacity (mg m^2^)** | **Reference** |
| --- | --- | --- | --- |
| Activated carbon | 7.5 | 0.067 | This work |
| PSP_10:90:1.000_ | 7.5 | 0.140 | This work |
| PSP_20:80:1.000_ | 7.5 | 0.153 | This work |
| PSP_30:70:1.000_ | 7.5 | 0.210 | This work |
| PSP_40:60:1.000_ | 7.5 | 0.355 | This work |
| PSP_40:60:0.250_ | 7.5 | 0.333 | This work |
| PSP_40:60:0.500_ | 7.5 | 0.252 | This work |
| PSP_40:60:1.500_ | 7.5 | 0.322 | This work |
| Activated carbon | 100.0 | 0.075 | 1 |
| Activated carbon | 500.0 | 0.159 | 2 |
| Activated carbon | 0.5 | 0.064 | 3 |
| Activated carbon | 120.0 | 0.216 | 4 |

PSP_X:Y:Z_ = X%[DIB]:Y%[S_8_]:Zg[NaCl].

1. K. K. Beltrame, A. L. Cazetta, P. S. C. de Souza, L. Spessato, T. L. Silva and V. C. Almeida, Ecotox Environ Safe, 2018, 147, 64-71.

2. H. G. Zanella, L. Spessato, G. K. P. Lopes, J. T. C. Yokoyama, M. C. Silva, P. S. C. Souza, A. Ronix, A. L. Cazetta and V. C. Almeida, J Mol Liq, 2021, 340.

3. V. Diniz, G. Rath, S. Rath, L. S. Araujo and D. G. F. Cunha, Environ Sci Pollut R, 2022, 29, 42185-42200.

4. G. Labuto, A. P. Carvalho, A. S. Mestre, M. S. dos Santos, H. R. Modesto, T. D. Martins, S. G. Lemos, H. D. T. da Silva, E. N. V. M. Carrilho and W. A. Carvalho, Sustain Chem Pharm, 2022, 28.

**LIST OF EQUATIONS**

*Kinetics models*

| $q_{i} = q_{\mathrm{ads}} (1 - e^{-k_{1}t})$ | Pseudo-first order model |
| --- | --- |
| $q_{i} = \frac{q_{\mathrm{ads}}^{2} k_{2} t}{1 + k_{2} q_{\mathrm{ads}} t}$ | Pseudo-second model |
| $q_{i} = \frac{1}{\beta} Ln (1 + \alpha\beta t)$ | Elovich model |
| $q_{i} = k_{i} \sqrt{t} + C$ | Intraparticle diffusion model |
| $\ln\left( 1-\frac{i}{q_{ads}} \right)=-k_{\mathrm{fd}} t$ | Film diffusion model |

Where q_i_ (mg g^-1^) represents the adsorption amount at the time of t (min), q_ads_ (mg g^-1^) is the adsorption potential and t is time (min). k_1_ (min^-1^)), k_2_ (g mgmin^-1^), k_i_ (mg g^−1^ min^−0,5^) are the adsorption rate constants of the pseudo-first-order, pseudo-second order and Intraparticle diffusion model, respectively. C is a constant involving the liquid film thickness. α (mg g^-1^ min-1) and β (mg g^-1^) are the parameters of the Elovich model.

*Isotherm models*

| $q_{i} = \frac{q_{ads} K_{L} C_{e}}{1 + K_{L} C_{e}}$ | Langmuir model |
| --- | --- |
| $q_{i} = K_{F} C_{e}^{\frac{1}{n}}$ | Freundlich model |
| $q_{i}= \frac{K_{RP} C_{e}}{1 + \alpha_{RP} C_{e}^{g}}$ | Redlich-Peterson model |
| $q_{i} = \frac{q_{ads} \left( K_{s} C_{e} \right)^{n}}{\left( K_{s} C_{e} \right)^{n} + 1}$ | Sips model |
| $Ln \left( q_{i} \right)= - k_{DR} \varepsilon^{2}+Ln \left( q_{ads} \right)$ | Dubinin-Radushkevich model |
| $\varepsilon=RT Ln \left( 1+ \frac{1}{\frac{C_{e}}{C_{s}}} \right)$ |  |
| $E= \frac{1}{\sqrt{2 k_{DR}}}$ |  |

Where K_L_ (L mg^-1^), K_F_ ((mg/g)/(mg/L)^n^), and K_RP_ (L mg^-1^), and K_S_ (L mg^-1^) are the Langmuir, Freundlich, Redlich-Peterson, and Sips model constants, C_e_ (mg L^-1^) is the concentration at equilibrium, 1/n is a constant related to the adsorption strength and n represents the inhomogeneity of the adsorbent. The closer the value of n is to 1, the more uniform the adsorbent surface is ^33^. $\alpha_{RP}$ ((mg L^-1^)^g^) and g are also constant of the Redlich- Peterson model.

KDR (mol^2^ kJ^-2^) is the constant of the Dubinin-Radushkevich model. ε (kJ mol^-1^) is the Polanyi potential. C_s_ is the solubility of caffeine in water at 25ºC (21.7 g L^-1^). E is the free energy (kJ mol^-1^).

*Thermodynamics models*

| $Ln \left( K_{2} \right)= - \frac{E_{a}}{RT}+Ln (A)$ | Arrhenius model |
| --- | --- |
| $Ln \left( \frac{K_{2}}{T} \right)= - \frac{\Delta H^{\#}}{RT}+ \left( Ln\left( \frac{k_{b}}{h} \right)+\frac{\Delta S^{\#}}{R} \right)$ | Eyring model |

Where A was the Arrhenius constant, R is the gas constant (8.314 J mol^-1^ K^-1^) and T is the temperature (K). $\Delta H^{\#}$ is the standard enthalpy (J mol^-1^), $\Delta S^{\#}$ is the entropy of activation (J mol^-1^ K^-1^), kb is the Boltzman constant (1.3807 × 10^− 23^ J K^− 1^), and h is the lank constant (6.6261 × 10^− 34^ Js).
